# A timeline of oligodendrocyte death and proliferation following experimental subarachnoid hemorrhage

**DOI:** 10.1111/cns.13812

**Published:** 2022-02-11

**Authors:** Kang Peng, Sravanthi Koduri, Fenghui Ye, Jinting Yang, Richard F. Keep, Guohua Xi, Ya Hua

**Affiliations:** ^1^ Department of Neurosurgery University of Michigan Ann Arbor Michigan USA; ^2^ Department of Neurosurgery Xiangya Hospital Central South University Changsha China

**Keywords:** corpus callosum, lipocalin‐2, oligodendrocytes, subarachnoid hemorrhage, T2 hyperintensity

## Abstract

**Aims:**

White matter (WM) injury is a critical factor associated with worse outcomes following subarachnoid hemorrhage (SAH). However, the detailed pathological changes are not completely understood. This study investigates temporal changes in the corpus callosum (CC), including WM edema and oligodendrocyte death after SAH, and the role of lipocalin‐2 (LCN2) in those changes.

**Methods:**

Subarachnoid hemorrhage was induced in adult wild‐type or LCN2 knockout mice via endovascular perforation. Magnetic resonance imaging was performed 4 hours, 1 day, and 8 days after SAH, and T2 hyperintensity changes within the CC were quantified to represent WM edema. Immunofluorescence staining was performed to evaluate oligodendrocyte death and proliferation.

**Results:**

Subarachnoid hemorrhage induced significant CC T2 hyperintensity at 4 hours and 1 day that diminished significantly by 8 days post‐procedure. Comparing changes between the 4 hours and 1 day, each individual mouse had an increase in CC T2 hyperintensity volume. Oligodendrocyte death was observed at 4 hours, 1 day, and 8 days after SAH induction, and there was progressive loss of mature oligodendrocytes, while immature oligodendrocytes/oligodendrocyte precursor cells (OPCs) proliferated back to baseline by Day 8 after SAH. Moreover, LCN2 knockout attenuated WM edema and oligodendrocyte death at 24 hours after SAH.

**Conclusions:**

Subarachnoid hemorrhage leads to T2 hyperintensity change within the CC, which indicates WM edema. Oligodendrocyte death was observed in the CC within 1 day of SAH, with a partial recovery by Day 8. SAH‐induced WM injury was alleviated in an LCN2 knockout mouse model.

## Graphical abstract

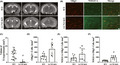

## 1 INTRODUCTION

Subarachnoid hemorrhage (SAH) has a high risk of morbidity and mortality, particularly in the initial few days following aneurysm rupture.^1^, ^2^ Aneurysm rupture can cause devastating increases in intracranial pressure, decreased cerebral blood flow, and exposure of brain parenchyma to toxic blood components (e.g., thrombin and red blood cell components), which further exacerbate early brain injury by potentiating brain edema, microcirculation dysfunction, blood–brain barrier (BBB) disruption, and white matter (WM) injury.^3^, ^4^, ^5^ White matter accounts for approximately 50% of the human brain volume and primarily contains oligodendrocytes and long axons.^6^ Studies have shown that WM injury is associated with cognitive impairment and motor dysfunction in patients who have suffered from SAH or ischemic stroke.^7^, ^8^, ^9^ Thus, it is essential to elucidate the mechanisms underlying WM injury in SAH, in the hopes that this will identify potential therapeutic targets.

Magnetic resonance imaging (MRI) has been utilized in SAH rodent models to evaluate post‐operative brain changes, which include basal clot, intraventricular hemorrhage, SAH‐induced acute hydrocephalus, and T2 hyperintensity lesions within the brain parenchyma^10^ and white matter.^11^ Recently, published studies found that ultra‐early (4 hours) thrombus formation can be detected on the T2* sequence and can persist for 24 hours.^12^, ^13^ Furthermore, a grading system classifying SAH severity based on MRI in mouse and rat models was established.^14^, ^15^ This study aimed to investigate WM MRI changes at different time points.

Oligodendrocytes, which include oligodendrocyte precursor cells (OPCs), immature oligodendrocytes, and mature oligodendrocytes (OLs), are a lineage of cells that are responsible for the generation of myelin in the central nervous system (CNS). Interestingly, they are also the most vulnerable cells of the CNS in pathological conditions.^16^ Oligodendrocyte death and myelin basic protein (MBP) loss have been described in hemorrhagic and ischemic stroke.^17^, ^18^, ^19^ The current study investigates dynamic changes in mature and immature oligodendrocytes following experimental SAH.

Our previous studies have demonstrated that experimental SAH in the ultra‐acute (4 hours) and early acute phases (24 hours) in the mouse model can induce significant corpus callosum (CC) edema and BBB disruption, which are attenuated in lipocalin‐2 (LCN2) knockout mice.^20^, ^21^ LCN2 is an acute phase protein involved in iron sequestration, apoptotic cell death, and regulation of cellular differentiation.^22^, ^23^ However, the mechanisms of how LCN2 may be involved in WM injury are still not completely understood. Here, we utilized a LCN2 knockout mouse model to investigate the role of LCN2 in the dynamic changes occurring in oligodendrocytes after SAH.

## 2 METHODS

### 2.1 Animal preparation and SAH induction

All animal protocols were approved by the University of Michigan Committee on the Use and Care of Animals. The study followed the ARRIVE guidelines for the reporting of in vivo animal experiments.^24^ Mice were raised in 12:12 light–dark conditions with free access to food and water. A total of 48 male wild‐type (WT) C57BL/6 mice (weight 22‐30 g, 3‐month‐old, Charles River Laboratories) and 11 male LCN2 knockout (LCN2^−/−^) mice with C57BL/6 background (3‐month‐old, University of Michigan Breeding Core) were used. Three WT mice and one LCN2^−/−^ mouse died after SAH induction and were excluded from this study.

Endovascular perforation was performed to induce SAH as previously reported.^20^ Briefly, mice initially underwent anesthetic induction with 5% isoflurane and were subsequently maintained at a constant rate of 1.5%. Core body temperature was maintained at 37.5°C for the entirety of the time that the mouse was anesthetized with the use of a controlled heating pad. A 5–0 blue monofilament suture with a blunted tip was inserted into the external carotid artery and carefully guided into the internal carotid artery until resistance was felt. The suture was then inserted an additional 1 mm to perforate the distal internal carotid artery. In the control group, the procedure was performed exactly as described above except for the perforation step, which was omitted.

### 2.2 Experimental groups

This study can be divided into four experimental groups. First, six adult WT mice underwent endovascular perforation followed by MRI at 4 hours after SAH induction. Second, a total of 31 adult WT mice underwent either endovascular perforation (*n* = 25) or sham (*n* = 6) operation followed by MRI at 4 hours and on Day 1 post‐procedure. Third, eight adult WT mice underwent endovascular perforation followed by MRI on Day 1 and Day 8 after SAH induction. Fourth, 10 adult LCN2^−/−^ mice underwent endovascular perforation followed by MRI at 24 hours. All mice were euthanized with intraperitoneal pentobarbital and their brains harvested for immunohistochemical assessment of oligodendrocyte cells. WT mice euthanized at 24 hours post‐procedure were utilized for localization of LCN2 expression.

### 2.3 Magnetic resonance imaging and WM T2 hyperintensity measurements

Magnetic resonance imaging was performed at 4 hours, on Day 1 and Day 8 after SAH using a 7.0 T Varian MRI scanner (Varian Inc) with acquisition of T2 fast spin‐echo and T2* gradient‐echo sequences with a field of view of 20 × 20 mm, matrix of 256 × 256 pixels, and 25 coronal slices (0.5 mm thick). Initial anesthesia induction with 5% isoflurane followed by a continuous rate of 1.5% isoflurane was administered during the MRI scans.^20^ The region of WM hyperintensity was identified as the volume of T2 hyperintensity within the corpus callosum and measured using ImageJ software by a blinded observer in all T2‐coronal slices.

### 2.4 Brain histology and hematoxylin and eosin staining

Mice were euthanized with pentobarbital (60 mg/kg IP) and perfused with 4% paraformaldehyde diluted in 0.1 M phosphate‐buffered saline (pH 7.4). Brains were harvested and then fixed in 4% paraformaldehyde for 24 hours at 4°C and then dehydrated in 30% sucrose for 4 days at 4°C. Brains were sectioned into 18‐μm thick slices using a cryostat. Hematoxylin and eosin (H&E) staining was performed, as previously described, to observe oligodendrocyte morphology by light microscopy.^25^

### 2.5 Immunofluorescence double labeling

Immunofluorescence double labeling was performed as previously described.^13^ The primary antibodies (Ab) were goat anti‐Olig2 (1:200 dilution, AF2418, R&D Systems), rabbit anti‐Olig2 (1:200 dilution, ab109186, Abcam), rabbit anti‐Nogo‐A (1:100 dilution, ab62024, Abcam), and goat anti‐LCN2 (1:100, AF1757, R&D Systems). The secondary Abs were Alexa Fluor 488‐conjugated donkey anti‐rabbit mAb (1:500 dilution, Invitrogen), Alexa Fluor 488‐conjugated donkey anti‐goat mAb (1:500 dilution, Invitrogen), Alexa Fluor 594‐conjugated donkey anti‐rabbit mAb (1:500 dilution, Invitrogen), and Alexa Fluor 594‐conjugated donkey anti‐goat mAb (1:500 dilution, Invitrogen). Negative controls omitted the primary antibodies.

Olig2 is a helix–loop–helix transcription factor found in all OPCs, immature oligodendrocytes, and mature oligodendrocytes. Thus, Olig2^+^ cell numbers reflect all oligodendrocytes. In contrast, Nogo‐A identifies only the mature phenotype. Cells which were Nogo‐A^−^/Olig2^+^ cells were subclassified as oligodendrocyte precursor cells/immature oligodendrocytes.^16^, ^26^, ^27^

### 2.6 Cell counting

**FIGURE 1:**
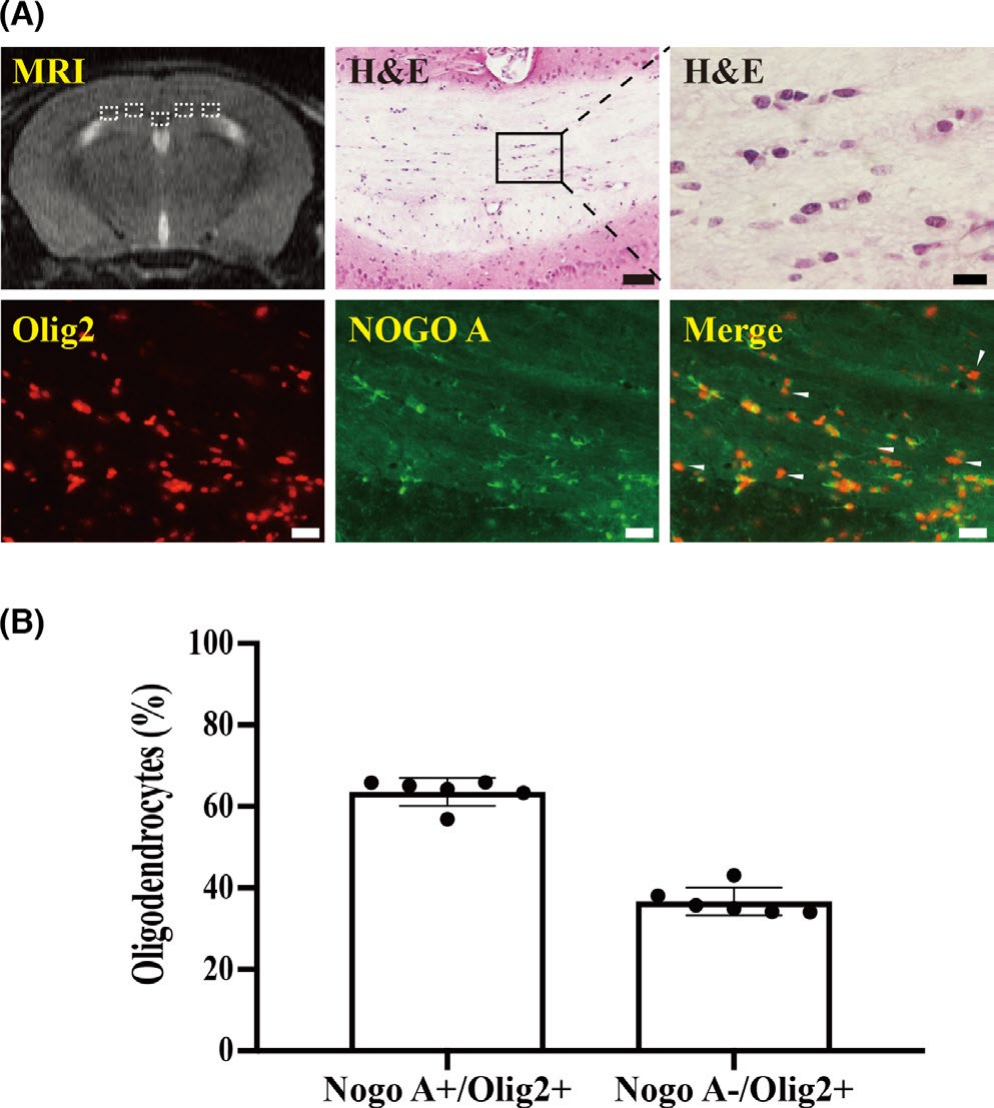
Morphology and quantification of oligodendrocytes within the corpus callosum of sham mice. (A) T2‐weighted MRI showing the regions (white boxes) selected for five high magnification images. Representative H&E images showing oligodendrocytes in the CC. Lower magnification, scale bar = 50 μm; higher magnification, scale bar = 10 μm. Representative immunofluorescence double labeling for Olig2 and Nogo‐A in the CC of the sham group. Olig2^+^ cells were considered as lineage oligodendrocytes; Nogo‐A^+^/Olig2^+^ cells were considered as OLs; Nogo‐A^−^/Olig2^+^ cells (white arrowheads) were considered as immature oligodendrocytes/OPCs. Scale bar = 20 μm. (B) Percentile of Nogo‐A^+^/Olig2^+^ cells, and Nogo‐A^−^/Olig2^+^ cells in Olig2^+^ cells. Values are mean ± SD, *n* = 6

Oligodendrocytes were quantified in five high‐power images (×40 magnification) that were obtained from the CC area represented in Figure 1A. Numbers of oligodendrocytes are hereafter presented as cells per square millimeter. All analyses were performed using ImageJ by a blinded observer.

### 2.7 Statistical analysis

Statistical analysis was performed using Prism 8 software. Presence of a normal distribution was determined using the Kolmogorov–Smirnov test. Unpaired student *t*‐test and ordinary one‐way ANOVA with Tukey's multiple comparisons test were used for data of normal distribution (represented as means ± SD). Mann–Whitney *U*‐test, Wilcoxon matched pair test, and nonparametric Kruskal–Wallis test with Dunn's multiple comparisons test were used for data of non‐normal distribution (represented as median). Significant differences were considered as *p* < 0.05.

## 3 RESULTS

Mortality rates were 0% (0/6) in the 4 hours group, 7% (2/27) in the 24 hours WT group, 11% (1/9) in the 8 day WT group, and 9% (1/11) in LCN2^−/−^ mice after SAH induction. There were no deaths in the sham group.

### 3.1 Morphology and classification of oligodendrocytes within the corpus callosum of sham mice

Five high magnification images were obtained from the regions depicted by the white boxes in Figure 1A on MRI for evaluation of oligodendrocytes. H&E staining shows oligodendrocytes in the corpus callosum in the sham group (Figure 1A). Immunofluorescence was performed in sham mice to quantify the total number of oligodendrocytes (Olig2^+^ cells), mature oligodendrocytes (Nogo‐A^+^/Olig2^+^ cells), and immature oligodendrocytes/OPCs (Nogo‐A^−^/Olig2^+^ cells; Figure 1A). Figure 1B shows the numerical breakdown of the two groups of cells making up all Olig2+ oligodendrocytes with immature oligodendrocytes/OPCs composing ~37% of all oligodendrocytes, and the remainder being mature oligodendrocytes.

### 3.2 Corpus callosum T2 hyperintensity was observed at 4 hours, worsened on Day 1, and reduced by Day 8 after SAH

**FIGURE 2:**
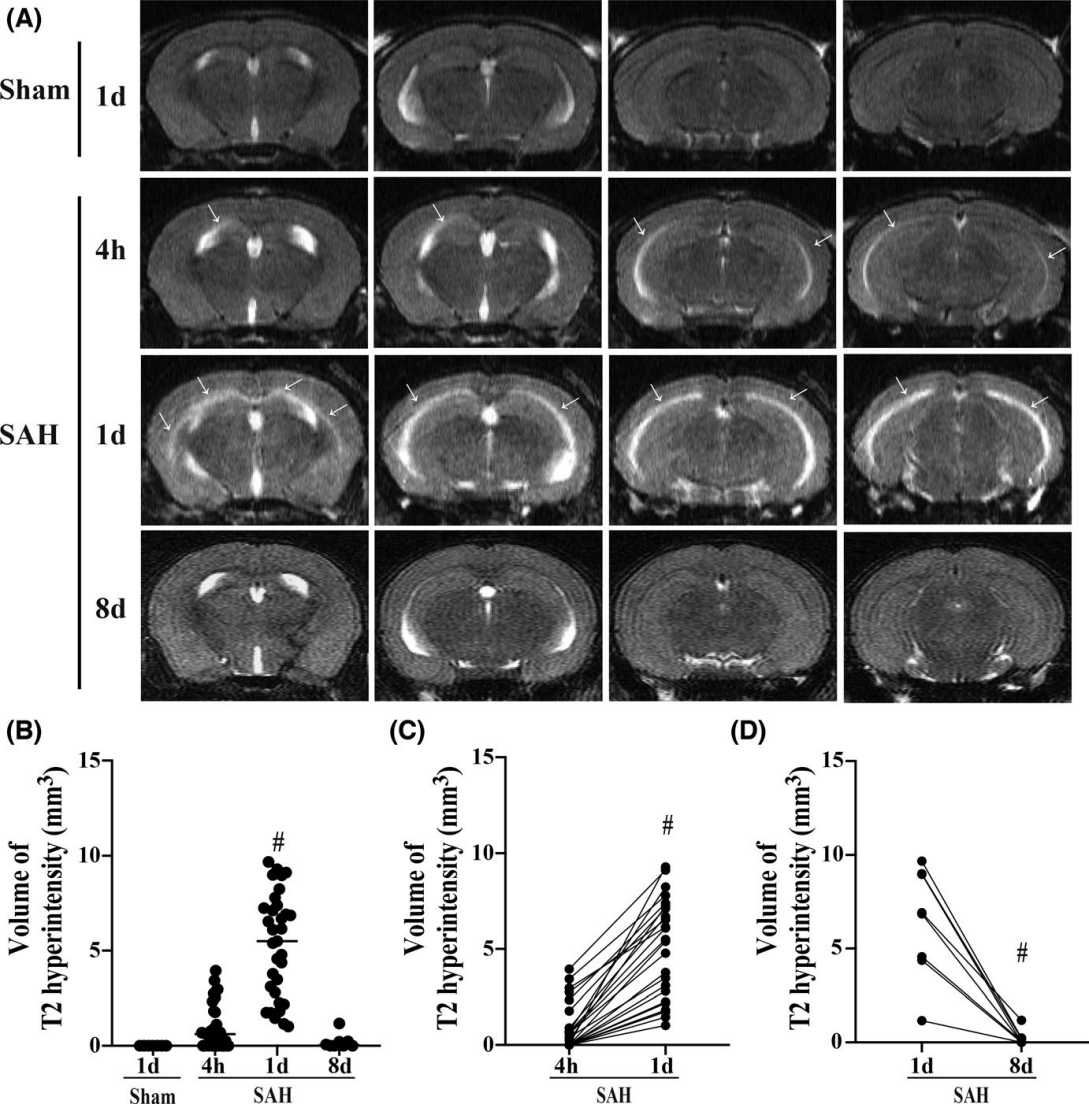
Corpus callosum T2 hyperintensity was observed at 4 hours, worsened on Day 1, and reduced by Day 8 after SAH. (A) Representative T2‐weighted MRI scans showing the area of T2 hyperintensity area at 4 hours, on Day 1, and Day 8 after SAH or Day 1 after sham operation in WT mice. White arrow indicates the region of T2 hyperintensity. (B) Quantification of the volume of T2 hyperintensity in the CC at 4 hours (*n* = 31), on Day 1 (*n* = 33), and Day 8 (*n* = 8) after SAH or sham operation (*n* = 6). Values as median; ^#^ *p* < 0.05 vs. sham. (C) Quantification of the volume of T2 hyperintensity in the CC of each individual mouse at 4 hours and on Day 1 after SAH. Values as median; *n* = 25; ^#^ *p* < 0.05. (D) Quantification of the volume of T2 hyperintensity in the CC of each individual mouse on Day 1 and Day 8 after SAH. Values as median; *n* = 8; ^#^ *p* < 0.05

Our previous studies have illustrated that SAH induces CC T2 hyperintensity and BBB disruption at 4 and 24 hours after SAH induction.^11^, ^20^, ^21^ The current study also found CC T2 hyperintensity at 4 hours (median = 0.62 mm^3^; *n* = 31) and Day 1 (median = 5.50 mm^3^; *n* = 33), with no hyperintensity in the sham group (median = 0 mm^3^; *n* = 6). A Kruskal–Wallis test showed that the difference between different time points was statistically significant, H (3) = 49.80, *t* < 0.05, and Dunn's multiple comparison test was used to compare pairs of groups. The difference between Day 1 and sham group was significant (Z = 4.844, *p* < 0.05; Figure 2A,B). Additionally, compared to imaging at 4 hours, the CC T2 hyperintensity volume at 24 hours was significantly increased in each individual mouse (Day 1: median = 5.40 mm^3^; 4 hours: median = 0.25 mm^3^). A Wilcoxon matched pairs signed rank test was conducted showing statistical significance (*n* = 25, *p* < 0.05; Figure 2C). Interestingly, the CC T2 hyperintensity volume was markedly reduced by Day 8 compared to Day 1 after SAH induction (Day 8: median = 0.04 mm^3^; Day 1: median = 6.88 mm^3^; *n* = 8, *p* < 0.05; Figure 2A,D). There was no significant difference in T2 hyperintensity between mice at Day 8 after SAH and the sham group (SAH: median = 0.04 mm^3^, *n* = 8; sham: median = 0 mm^3^, *n* = 6, Z = 0.66, *p* > 0.05; Figure 2A,B).

### 3.3 Oligodendrocytes die acutely after SAH, but immature oligodendrocyte/OPC recovery was observed by 8 days after SAH

**FIGURE 3:**
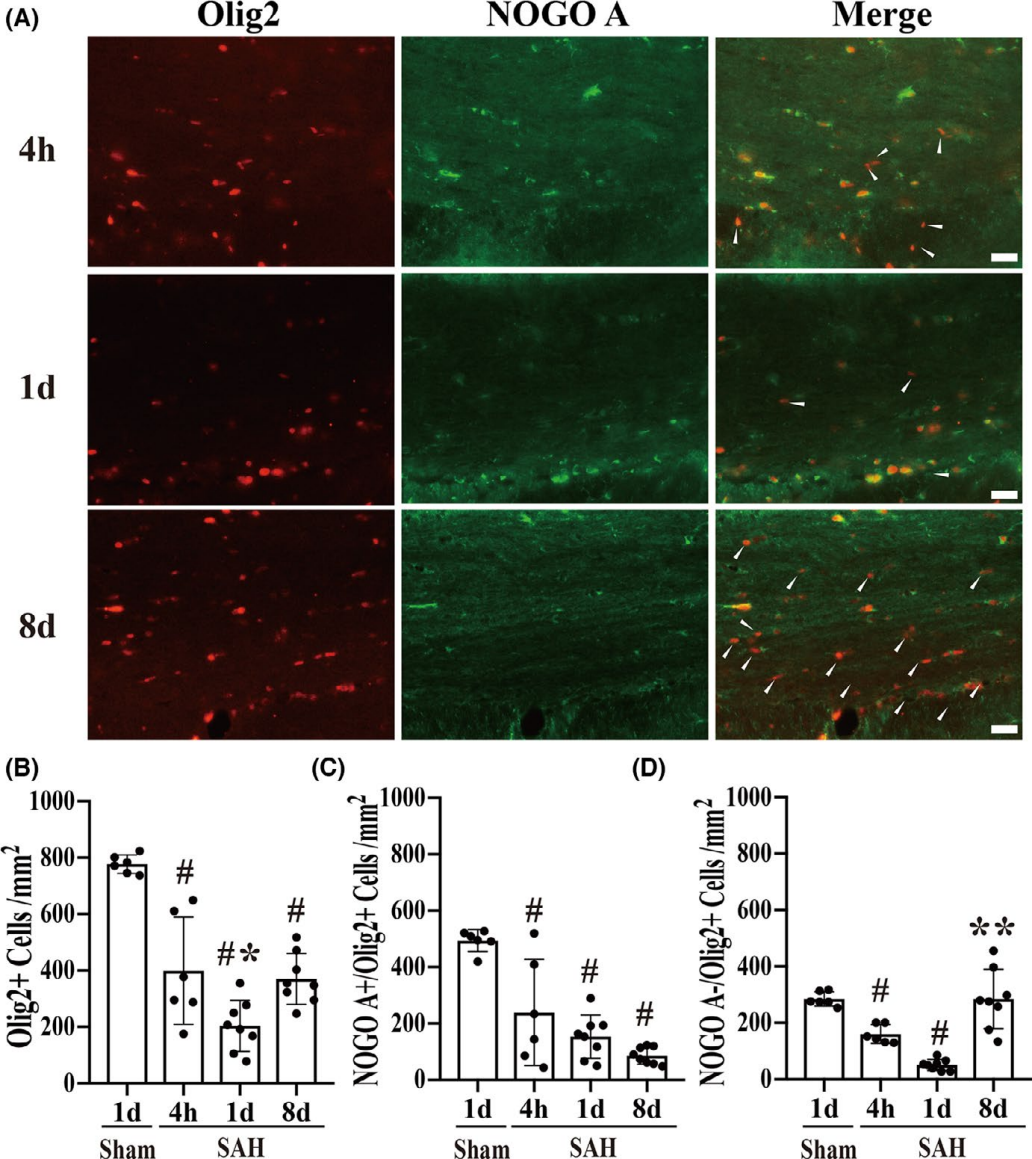
All OLs, immature oligodendrocytes, and OPCs died acutely after SAH but immature oligodendrocytes/OPCs recovery was observed by 8 days after SAH. (A) Representative immunofluorescence double labeling for Olig2 and Nogo‐A in the CC at 4 hours, on Day 1, and Day 8 after SAH. Scale bar = 20 μm. Quantification of (B) Olig2^+^ cells, (C) Nogo‐A^+^/Olig2^+^ cells, and (D) Nogo‐A^−^/Olig2^+^ cells (white arrowheads) at 4 hours (*n* = 6), on Day 1 (*n* = 8), and Day 8 (*n* = 8). Values as mean ± SD; ^#^ *p* < 0.05 vs. sham group; *p < 0.05 vs. the SAH 4h and 8d groups; **p < 0.05 vs SAH 4 hours and 1 days groups. Multiple comparisons were made using a Tukey's test. In (B), the differences in Olig2^+^ cells from sham controls were 378 cells/mm^2^ (95% confidence interval (CI) [199, 556]) at 4 hours, 573 cells/mm^2^ (95% CI [406, 740]) at 1 day, and 407 cells/mm^2^ (95% CI [240, 574]) at 8 days post‐SAH. The difference between Day 1 and 4 hours after SAH was 196 cells/mm^2^ (95% CI [29, 362]) and between Days 1 and 8 was 166 cells/mm^2^ (95% CI [12, 321]). In (C), the difference in mature Nogo‐A^+^/Olig2^+^ oligodendrocytes from sham controls was 255 cells/mm^2^ (95% CI [98, 412]) at 4 hours and 341 cells/mm^2^ (95% CI [194, 488]) at 1 day and 408 cell/mm^2^ (95% CI [261, 556]) at 8 days after SAH. In (D), the difference in immature Nogo‐A^+^/Olig2^+^ oligodendrocytes/OPCs from sham controls was 123 cells/mm^2^ (95% CI [26, 219]) at 4 hours and 232 cells/mm^2^ (95% CI [142, 323]) at 1 day after SAH induction. There was more cell loss on Day 1 compared to 4 hours post‐SAH: difference 109 cells/mm^2^ (95% CI [19, 200]) but a recovery by Day 8 (difference vs. Day 1, 234 cells/mm^2^, 95% CI [150, 317])

Next, lineage oligodendrocytes (Olig2^+^), mature oligodendrocytes (Nogo‐A^+^/Olig2^+^), and immature oligodendrocytes/oligodendrocyte progenitor cells (Nogo‐A^−^/Olig2^+^) were evaluated. Both Olig2^+^ lineage oligodendrocytes and Nogo‐A^+^/Olig2^+^ mature oligodendrocytes were markedly decreased at 4 hours, on Day 1 and Day 8 after SAH, compared to the sham group (Figure 3A). Nogo‐A^−^/Olig2^+^ immature oligodendrocytes/OPCs were also markedly decreased at 4 hours and on Day 1 after SAH compared to the sham group. Interestingly, however, these cells appeared to recover by Day 8 after SAH (Figure 3A).

When Olig2^+^ lineage oligodendrocytes were quantified, there was a significant difference among the groups (Figure 3B; F (3,24) = 30.84, *p* < 0.05). Post hoc comparisons using a Tukey's test (Figure 3B) revealed there was a reduction in these cell from 778 ± 33 cells per mm^2^ (*n* = 6) in the sham‐operated group to 400 ± 191 (*n* = 6), 205 ± 91 (*n* = 8), and 371 ± 89 (*n* = 8) cells per mm^2^ at 4 hours, 1 day, and 8 days after SAH, respectively. On Day 1, there was greater Olig2^+^ lineage oligodendrocyte loss than observed at 4 hours after SAH induction with some evidence of recovery by Day 8 (Figure 3B).

For Nogo‐A^+^/Olig2^+^ mature oligodendrocytes, there was also a significant difference between groups (Figure 3C; F (3,24) = 21.61, *p* < 0.05). Post hoc comparisons using a Tukey's test (Figure 3C) revealed a significant reduction at 4 hours (240 ± 189 cells per mm^2^; *n* = 6) and on Day 1 (154 ± 79 per mm^2^; *n* = 8) after SAH induction, compared to the sham group (495 ± 39 per mm^2^, Figure 3C). On Day 8 after SAH, the Nogo‐A^+^/Olig2^+^ mature oligodendrocytes declined further (86 ± 30 per mm^2^; *n* = 8) compared to sham group (Figure 3C).

For Nogo‐A^−^/Olig2^+^ immature oligodendrocytes/OPCs, there was also a significant difference between groups (Figure 3D; F (3,24) = 25.59, *p* < 0.05). Post hoc comparisons using a Tukey's test (Figure 3D) revealed a significant reduction at 4 hours (160 ± 33 per mm^2^; *n* = 6) and on Day 1 (51 ± 20 per mm^2^; *n* = 8) after SAH induction compared to sham controls (285 ± 25 per mm^2^; *n* = 6). There was more Nogo‐A^−^/Olig2^+^ cell loss on Day 1 after SAH induction in comparison with 4 hours post‐SAH (Figure 3D). Interestingly, however, on Day 8 post‐SAH, the number of Nogo‐A^−^/Olig2^+^ cells rose significantly compared to 1 day post‐SAH (285 ± 105 per mm^2^ on Day 8 (Figure 3D). Indeed, in certain mice, they increased to a level equivalent to that in the sham group (Figure 3D). These data imply that immature oligodendrocytes/OPCs proliferate at some time point between 1 day and 8 days following SAH. At Day 8, these cells were 77% of all oligodendrocytes (compared to 37% in sham‐operated mice).

### 3.4 LCN2 colocalizes with both Olig2 and Nogo‐A at 24 hours after SAH induction

**FIGURE 4:**
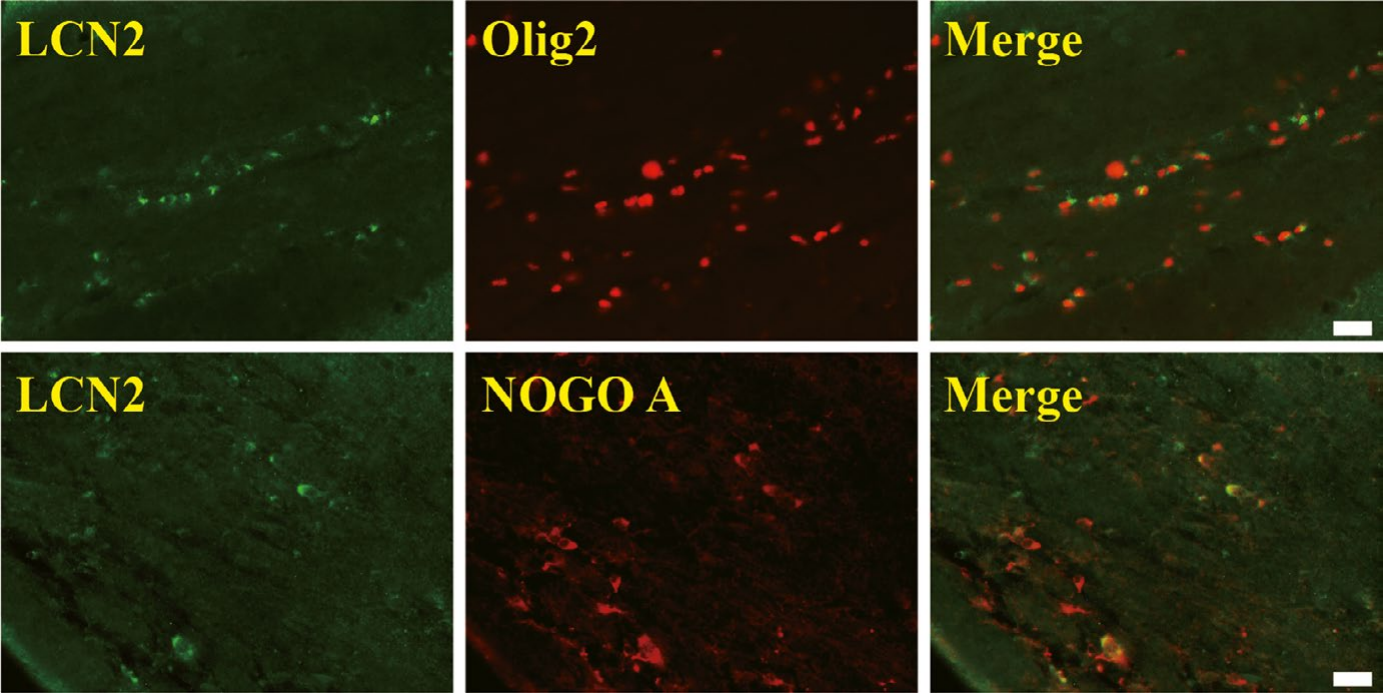
Lipocalin‐2 colocalizes with both Olig2 and Nogo‐A 24 hours after SAH induction. Immunofluorescence double labeling for LCN2 with Olig2 or Nogo‐A shows that both lineage oligodendrocytes and OLs express LCN2. Scale bar = 20 μm

Immunofluorescence double labeling for LCN2 with Olig2 or Nogo‐A was performed to determine whether oligodendrocytes express LCN2. Double labeling showed that LCN2 colocalizes with both Olig2^+^ cells and Nogo‐A^+^ cells (Figure 4).

### 3.5 LCN2 deficiency attenuates corpus callosum T2 hyperintensity and oligodendrocyte death after SAH induction

**FIGURE 5:**
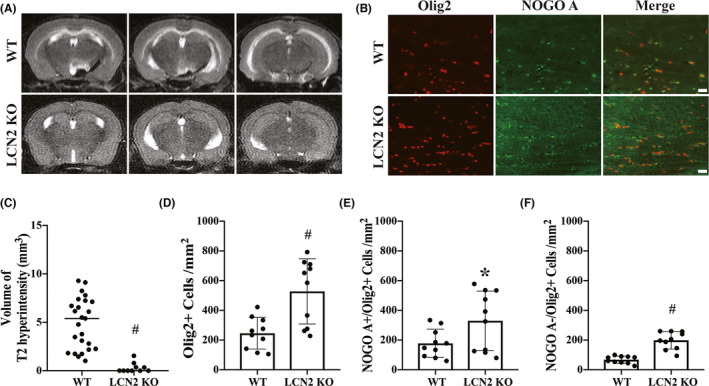
Lipocalin‐2 deficiency attenuates corpus callosum T2 hyperintensity and oligodendrocyte death after SAH induction. (A) Representative T2‐weighted MRI scans showing the region of T2 hyperintensity on Day 1 after SAH in WT (*n* = 25) and LCN2^−/−^ mice (*n* = 10). Values as median; ^#^ *p* < 0.01. (B) Representative immunofluorescence double labeling for Olig2 and Nogo‐A in the CC on Day 1 after SAH in WT and LCN2^−/−^ mice. Scale bar = 20 μm. Quantification of (C) volume of T2 hyperintensity, (D) Olig2^+^ cells, (E) Nogo‐A^+^/Olig2^+^ cells, and (F) Nogo‐A^−^/Olig2^+^ cells on Day 1 after SAH in WT (*n* = 10) and LCN2^−/−^ (*n* = 10) mice. Values as mean ± SD; ^#^ *p* < 0.01; *p < 0.05

To further examine whether LCN2 deficiency affects CC T2 hyperintensity and oligodendrocyte death after SAH induction, LCN2^−/−^ mice underwent endovascular perforation to generate the SAH model. Compared to the WT mice, LCN2^−/−^ mice had almost negligible T2 hyperintensity on Day 1 after endovascular perforation (LCN2^−/−^ mice: median = 0 mm^3^, *n* = 10; WT mice: median = 5.396, *n* = 25; U = 2, *p* < 0.01; Figure 5A,C). Olig2 and Nogo‐A immunofluorescence double labeling was performed and was consistent with the diminished T2 hyperintensity; more Olig2^+^ lineage oligodendrocytes (528 ± 220 vs. 246 ± 106 per mm^2^ in WT mice; *n* = 10; *t* (18) = 3.656, *p* < 0.01; Figure 5B,D) and Nogo‐A^+^/Olig2^+^ mature oligodendrocytes (330 ± 200 vs. 178 ± 96 per mm^2^ in WT mice; *t* (18) = 2.170, *p* < 0.05; Figure 5B,E) survived in the LCN2^−/−^ mice on Day 1 compared to WT mice after SAH induction. Additionally, more Nogo‐A^−^/Olig2^+^ immature oligodendrocytes/OPCs remained in the LCN2^−/−^ mice than in the WT mice at 24 hours after SAH induction (198 ± 59 vs. 68 ± 24 per mm^2^ in WT mice; *t* (18) = 6.476, *p* < 0.01; Figure 5B,F). Taken together, these data suggest that LCN2 plays a critical role in oligodendrocyte loss and WM injury after SAH.

## 4 DISCUSSION

Based on the data presented above, there are four major findings: (1) endovascular perforation induced experimental SAH causes CC T2 hyperintensity in the ultra‐acute phase (4 hours) that worsens in the early acute phase (1 day) and improves by the late acute phase (8 days); (2) SAH caused significant oligodendrocyte death in the ultra‐acute and acute phases which, for mature oligodendrocytes, persisted into the late acute phase; (3) in contrast, there was evidence of immature oligodendrocyte/OPC proliferation between Day 1 and Day 8; (4) LCN2 knockout exerts a protective role, reducing CC edema and oligodendrocyte death during the early acute phase.

In this study, we focused on the CC and delineated the temporal changes in CC T2 hyperintensity found on MRI after SAH induction by endovascular perforation. Magnetic resonance imaging is widely accepted as a reliable non‐invasive technique to evaluate early brain injury in hemorrhagic and ischemic stroke.^10^, ^28^, ^29^ Our prior studies have reported that WM T2 hyperintensity formation occurs in about 57% of WT mice at 4 hours, and in all WT mice by 1 day after SAH.^20^, ^21^ To add on to this prior literature, we further examined the dynamic change in each individual mouse at 4 hours and on Day 1 and found that not only does the CC T2 hyperintensity increase on Day 1, but each mouse experiences an increase in CC T2 hyperintensity between the 4 hours and 1 day time points after SAH. Furthermore, the changes observed from Day 1 to Day 8 are consistent with our previous report.^11^ Knowing the temporal change in CC T2 hyperintensity is quite important in determining the timeframe of maximal injury and optimizing the window for a therapeutic intervention and thereby, prompting a better outcome.

Oligodendrocyte precursor cells are a population of cells that maintain WM homeostasis and participate in long‐term WM repair after injury.^30^ OLs differentiate from OPCs and are responsible for producing myelin to form the insulating sheath of axons. Oligodendrocytes have long been thought to be the most vulnerable cells in the CNS in pathological conditions.^16^ During a hemorrhagic stroke, blood components leak into the parenchyma from vessels, setting off a series of stress‐inducing changes, including oxidative stress, cell death, and iron toxicity.^31^, ^32^, ^33^, ^34^ During an ischemic stroke, though there may be minimal blood extravasating into the parenchyma, damage is induced by reactive oxygen species, cytotoxic edema, and cell depolarization.^35^ Evidence shows that neuroinflammation and excitotoxicity contribute to early WM injury after SAH, which has components of both hemorrhagic and ischemic stroke.^36^ This study demonstrates the changes in lineage oligodendrocytes, OLs, and immature oligodendrocytes/OPCs at different phases after SAH and found that OLs decreased in number during all three phases. However, although immature oligodendrocytes/OPCs decreased during the ultra‐acute and early acute phases, they recovered in number by the late acute phase. Interestingly, these temporal changes in oligodendrocytes are consistent with the changes in T2 hyperintensity observed on MRI suggesting that oligodendrocyte death may contribute to CC edema. This is relevant from a clinical perspective as global cerebral edema is an independent risk factor for worse outcome after SAH.^37^

The underlying mechanism of SAH‐induced OL/OPC death is not clear. BBB disruption is known to play an important role in early brain injury.^38^ Our prior studies have indicated that BBB leakage correlates with white matter injury after SAH and LCN2^−/−^ has been shown to mitigate WM T2 hyperintensity and reduce BBB leakage^20^, ^21^ as well as reduce OL loss at 4 hours after SAH.^21^ The present study further discovered that LCN2^−/−^ reduced lineage oligodendrocyte and immature oligodendrocyte/OPC loss on Day 1 after SAH, not just that of the OLs. This suggests that LCN2 may be involved in oligodendrocyte death after SAH. LCN2 is an acute phase protein involved in various interconnected neuropathophysiological processes, which include exacerbation of neuroinflammation, cell death, and iron dysregulation.^22^, ^23^, ^39^ However, the detailed mechanism of how LCN2 may be involved in WM damage still needs further exploration. A recent study suggested that inhibition of ferroptosis alleviates SAH‐induced brain injury, including brain edema and neuronal death.^40^ Further studies are warranted to elucidate the role, if any, of ferroptosis in SAH‐induced OL/OPC death.

There are several limitations in this study. Only male mice were used in this study, and sex is known to be an important factor in prognosis.^25^, ^41^, ^42^ Therefore, more studies investigating sex differences are needed to create a complete picture. Only ultra‐acute, early acute, and late acute phases were examined. Long‐term experiments are necessary to gain a thorough understanding of the changes in oligodendrocytes and myelination patterns after SAH (e.g., can the increased proliferation of immature oligodendrocytes/OPCs between Days 1 and 8 eventually lead to more mature oligodendrocytes). Furthermore, this study only demonstrates changes in oligodendrocyte numbers while other biological endpoints, such as neurotransmission conductance in white matter, were not investigated. Finally, cognitive and functional assessments were not performed, which, when combined with a long‐term experiment, would provide a greater understanding of the role of oligodendrocytes in cognitive outcomes and recovery after SAH.^43^

## 5 CONCLUSIONS

In conclusion, CC T2 hyperintensity and oligodendrocyte death occur during the ultra‐acute (4 hours) and early acute phases (1 day) after SAH with a reduction in CC T2 hyperintensity and recovery of immature oligodendrocytes/oligodendrocyte precursor cells during the late acute phase (8 days). The temporal pattern of oligodendrocyte death is consistent with CC T2 hyperintensity formation suggesting oligodendrocytes participate in the production of CC edema, at least in part. LCN2 deficiency reduced CC hyperintensity and oligodendrocyte death during the acute phase after SAH.

## CONFLICT OF INTEREST

The authors declare there is no conflict of interest.

## Data Availability

All data in this article can be provided by the corresponding author Dr. Guohua Xi, upon reasonable request.
